# Baculovirus-free insect cell expression system for high yield antibody and antigen production

**DOI:** 10.1038/s41598-020-78425-9

**Published:** 2020-12-07

**Authors:** Janin Korn, Dorina Schäckermann, Toni Kirmann, Federico Bertoglio, Stephan Steinke, Janyn Heisig, Maximilian Ruschig, Gertrudis Rojas, Nora Langreder, Esther Veronika Wenzel, Kristian Daniel Ralph Roth, Marlies Becker, Doris Meier, Joop van den Heuvel, Michael Hust, Stefan Dübel, Maren Schubert

**Affiliations:** 1 Department of Biotechnology, Technische Universität Braunschweig, Spielmannstraße 7, 38106 Braunschweig, Germany; 2 Center of Molecular Immunology, PO Box 16040, 11300 Havana, Cuba; 3 Department Structure and Function of Proteins, Helmholtz-Centre for Infection Research, Inhoffenstraße 7, 38124 Braunschweig, Germany; 4 Present Address: Medical Faculty, Carl Ludwig Institute for Physiology, Universität Leipzig, Liebigstraße 27, 04103 Leipzig, Germany; 5 Present Address: Department Vaccinology and Applied Microbiology, Helmholtz Centre for Infection Research, Inhoffenstraße 7, 38124 Braunschweig, Germany

**Keywords:** Expression systems, Transfection, Biotechnology, Transfection

## Abstract

Antibodies are essential tools for therapy and diagnostics. Yet, production remains expensive as it is mostly done in mammalian expression systems. As most therapeutic IgG require mammalian glycosylation to interact with the human immune system, other expression systems are rarely used for production. However, for neutralizing antibodies that are not required to activate the human immune system as well as antibodies used in diagnostics, a cheaper production system would be advantageous. In our study, we show cost-efficient, easy and high yield production of antibodies as well as various secreted antigens including Interleukins and SARS-CoV-2 related proteins in a baculovirus-free insect cell expression system. To improve yields, we optimized the expression vector, media and feeding strategies. In addition, we showed the feasibility of lyophilization of the insect cell produced antibodies. Furthermore, stability and activity of the antibodies was compared to antibodies produced by Expi293F cells revealing a lower aggregation of antibodies originating from High Five cell production. Finally, the newly established High Five expression system was compared to the Expi293F mammalian expression system in regard of yield and costs. Most interestingly, all tested proteins were producible in our High Five cell expression system what was not the case in the Expi293F system, hinting that the High Five cell system is especially suited to produce difficult-to-express target proteins.

## Introduction

The global market for monoclonal antibodies used in therapy or diagnostics has grown over the past years and is estimated to reach US$132 billion dollar by 2023^1^. Most of these monoclonal antibodies are produced in mammalian expression systems. Thanks to process optimization production, costs decreased from US$300/g to under US$30/g at ideal condtions^2–4^. However, setting up an optimal production system with an optimized cell clone is time- and cost-intensive. For diagnostic antibodies this is not necessary thus fast transient plasmid-based production is the method of choice to produce research-scale quantities of antibodies^5^. In addition, diagnostic antibodies do not require mammalian glycosylation as they do not have to interact with the human immune system, allowing the use of alternative expression systems. Thus, insect cells are an ideal alternative to reduce efforts and cost, as they combine ease of culture (at 27 °C without requirement of CO_2_) with higher tolerance to osmolality of the medium, by-product concentration^6^ and cheaper media^7,8^.

Production of recombinant proteins in insect cells with baculovirus has a long history dating back to the mid 1980s^9–11^. Substantial optimization regarding handling, protein yield, deletion of proteases and other factors was achieved over time^12–14^. Yet, the baculovirus expression vector system (BEVS) still is not optimal for the production of secreted proteins as the virus infection negatively affects the secretory pathway of its host cells^15^. This impairs both yield and protein quality, in particular when the highly active but very late promoters polH or p10 are used^16^. This bottleneck is critical for production of antibodies and therefore only few attempts have been made to produce antibodies by BEVS^17–20^ as the resulting yield was rather low.

Recently, the plasmid-based production in insect cells without the use of baculovirus was reported^21–26^. Different protocols and expression vectors exist but in each case the expression vector is quite efficiently delivered by Polyethylenimine (PEI) and no baculovirus is required. Without baculoviral infection, the host cells maintain their original secretory pathway integrity, normal cell growth and high viability, resulting in a higher quality of the secreted protein. Our previous studies already demonstrated a higher protein yield of a secreted protein compared to BEVS with our plasmid-based High Five expression system^25^.

In this study, we investigated the potential of the plasmid-based High Five expression system for production of secreted proteins with a focus on antibody and Fc-fusion protein production. Hereto, we first optimized the expression vector for secreted target proteins. Secondly, we evaluated the most suitable time-point of harvest as secreted protein are supposed to accumulate over time in the cultivation media. Thirdly, we also tested whether media supplements increase expression of antibodies. After this optimization of the system towards secreted proteins, we confirmed the possibility to lyophilize antibodies produced in insect cells. Furthermore, we compared antibody quality in regard of stability and aggregation behaviour when produced in insect cells to those produced in the mammalian Expi293F cell system. Finally, we compared production yields of different secreted proteins in our insect cell expression system to those obtained using Expi293F cells.

## Results

### Vector design for secreted target proteins

It is widespread knowledge, that the expression vector design influences the yield of recombinant protein^27–29^. Therefore, different vector designs for high expression of secreted target proteins were compared. In particular, the influence of the Kozak sequence, the insertion of an intron inside the secretion signal and two different secretion signal peptides were tested resulting in four different expression vectors. To exclude the influence of the protein itself on these elements, the expression of two different IgG (EWE192-B5P-hIgG and TUN219-2C1-mIgG) and two scFv-Fc (TUN219-2C1-hFc and KRO65-A4-hFc) was evaluated with all designed vector variants.

**Figure 1:**
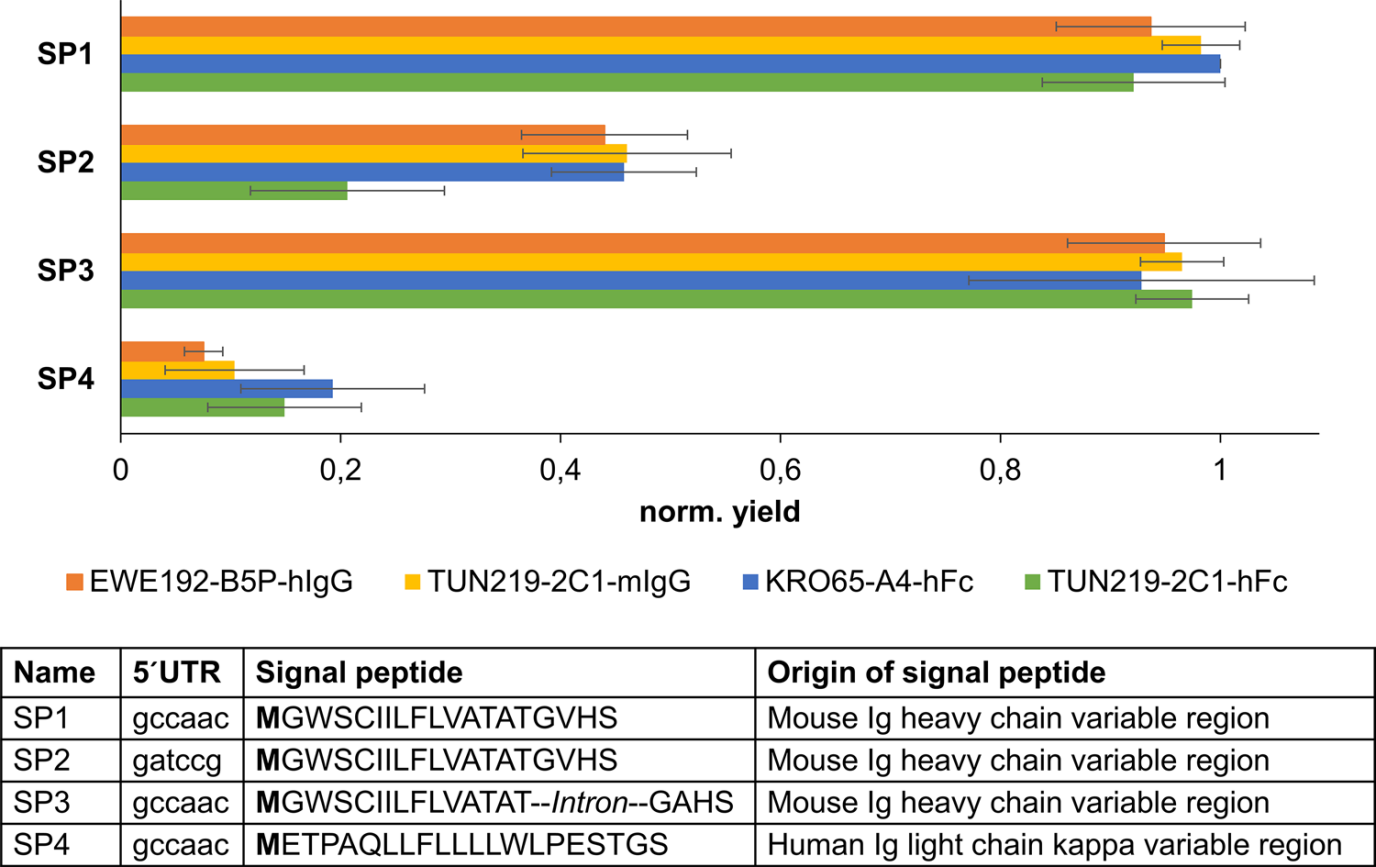
Normalized yield of two different IgG and two different scFv-Fc expressed by the different vectors employing different 5′UTR or signal peptides. The yields were normalized against the respective highest yield obtained with one of the four expression vectors. All experiments were performed four times; standard deviation is shown.

To determine the effect of 5′UTR on expression a 5′UTR similar to the mammalian Kozak (GCCAACatg) and a random 5′UTR (GATCCGatg) were compared (SP1 vs. SP2, Fig. 1). Remarkably, the expression level decreased by 50% up to 70% when the random 5′UTR was applied (SP2), underlining that indeed the 5′UTR has a drastic influence on expression. As it was shown in literature that introns might enhance expression in eukaryotic expression systems^30,31^, the effect of an intron of the mouse Immunoglobulin heavy chain variable region was also tested. However, no significant impact on expression yield was observed with or without the presence of the intron in High Five cells (SP1 vs SP3, Fig. 1). In contrast, the choice of signal peptide had again a drastic influence, decreasing yields below 20% (SP1 vs. SP4, Fig. 1).

In conclusion, expression vectors for secreted proteins should include the Kozak sequence as well as the signal peptide of mouse Ig heavy chain variable region.

### Determination of the optimal time point for harvest

**Figure 2:**
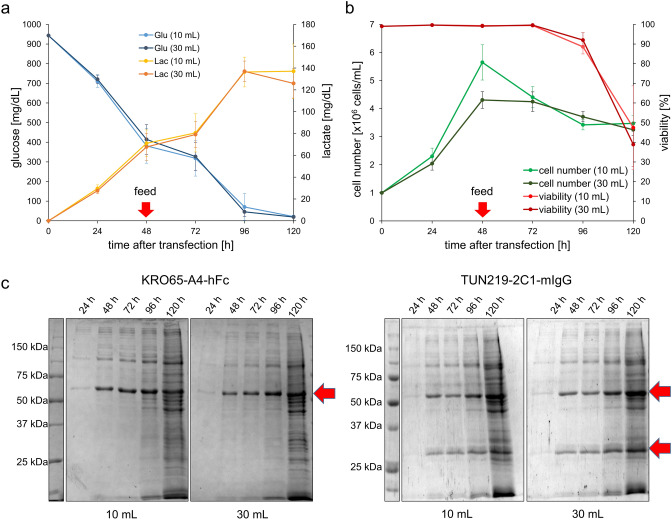
Cultivation parameters over 120 h. (**a**) Average Glucose and Lactate concentration in the media and (**b**) cell number and viability over time of IgG and scFv-Fc production (each done three times). (**c**) SDS–PAGE of the supernatant at the indicated days of the different production scales (10 mL or 30 mL) of KRO65-A4-hFc (~ 52.9 kDa) and TUN219-2C1-mIgG (Heavy chain ~ 51.5 kDa, light chain ~ 24.1 kDa). In total three SDS–PAGEs were grouped together for this figure, the original SDS–PAGEs are shown in Supplementary Data 1.

Harvesting after 72 h of expression has been described to be optimal for recombinant cytoplasmic proteins^32^. In contrast, secreted proteins can accumulate outside of the cell in the supernatant over a longer time period, assuming they are stable enough. Thus, a later time point of harvest seemed favourable. Yet, if cell viability drops too low, a vast amount of intracellular proteins will be released from dead cells, impairing downstream processing and increasing the risk of degradation by proteases. Therefore, the cultivation process of both a 10 mL and a 30 mL production of an IgG and an scFv-Fc was closely monitored over 120 h in respect of cell density, viability, production yield and glucose/lactate content of the media (Fig. 2).

For both proteins the max. protein yield was reached after 96–120 h while viability was still above 85% at 96 h decreasing to ~ 45% at 120 h. Reason for the rather drastic drop of viability after 120 h could be almost complete depletion of glucose after 96 h. After 120 h already a vast amount of additional- probably intracellular- proteins was visible on SDS–PAGE.

Interestingly, the production scale did not have a significant impact on glucose/lactate concentration, transfection efficacy or viability even if production vessels (30 mL tube vs. 125 mL Corning culture vessel) and also shaking speed (200 rpm vs. 110 rpm, respectively) differ*.* No significant differences were observed between the productions of scFv-hFc and mIgG.

In conclusion, 96 h after transfection was considered as the optimal time point of harvest for both target proteins as the yield nearly reached the maximum while avoiding the excessive contaminating proteins that appeared at later harvesting time points.

### Supplementation of the media

**Figure 3:**
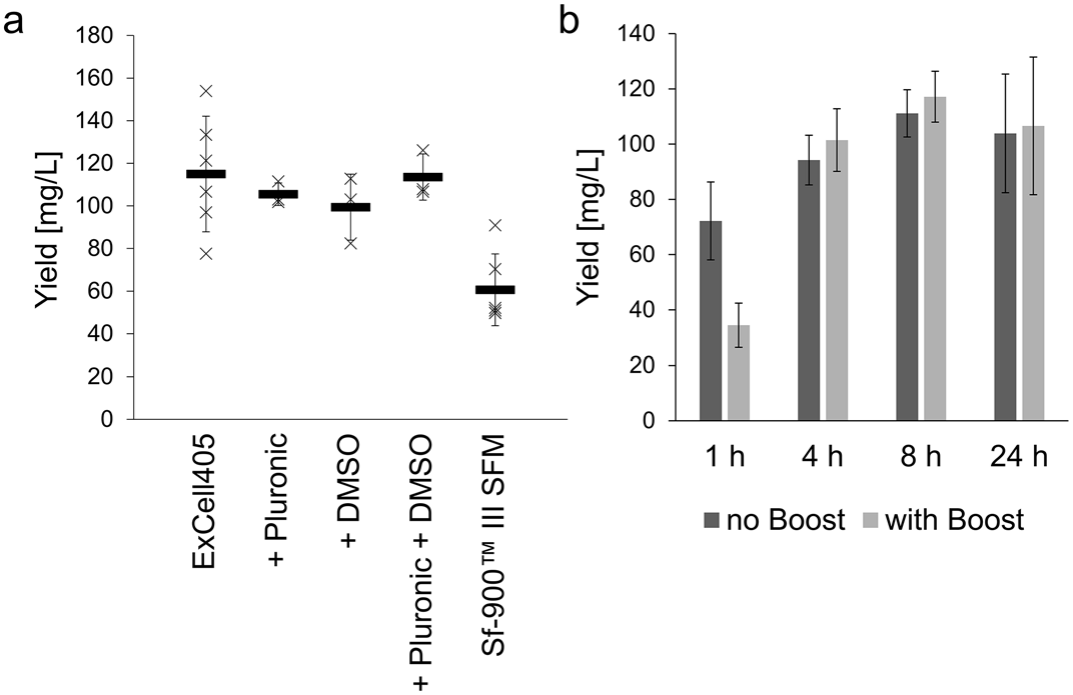
Effect of media and additives of the production of TUN219-2C1-hFc in High Five insect cells. (**a**) Comparison of production in different media (EX-CELL405 and Sf-900™ III SFM) and EX-CELL405 complemented with different supplements (0.1% Pluronic F68, 0.05% DMSO or both). (**b**) Evaluation of different time points for the first feeding after transfection with and without addition of 10% HyClone Cell Boost 6. All experiments were performed in quadruplicates; the standard deviation is indicated.

Different additives (Pluronic F68, DMSO), known to have an impact on transfection^7^, and different media, known to be compatible with this method^25^ (EX-CELL405 (Merck) and Sf-900™ III SFM (ThermoFisher)), were tested for their impact on expression (Fig. 3a). Neither addition of 0.1% extra Pluronic F68 (EX-CELL405 media already contains 0.1% Pluronic F68) nor 0.05% DMSO showed a significant impact on the yield. However, transfection efficacy was up to 10% higher in Sf-900™ III SFM media (data not shown) but contrary the scFv-Fc yield was significantly lower (~ 40%). Consequently, EX-CELL405 media was chosen for the further experiments.

Next, different time points for the first feeding after transfection were evaluated, as well as the addition of 10% HyClone Cell Boost 6 supplement (Cytiva), a supplement also used in the mammalian Expi293F system (Fig. 3b). The HyClone Cell Boost 6 inhibited transfection when added only 1 h after transfection, decreasing transfection efficiency and yield. Otherwise the Boost did not have a significant impact on production in insect cells. The best time point for the first feeding in general was between 4 and 24 h, indicating that the DNA-PEI uptake was mainly complete after ~ 4 h.

In conclusion, EX-CELL405 media on its own was optimal and none of the tested additives could increase the yield.

### Stability and activity of the antibodies produced in High Five compared to mammalian production

**Figure 4:**
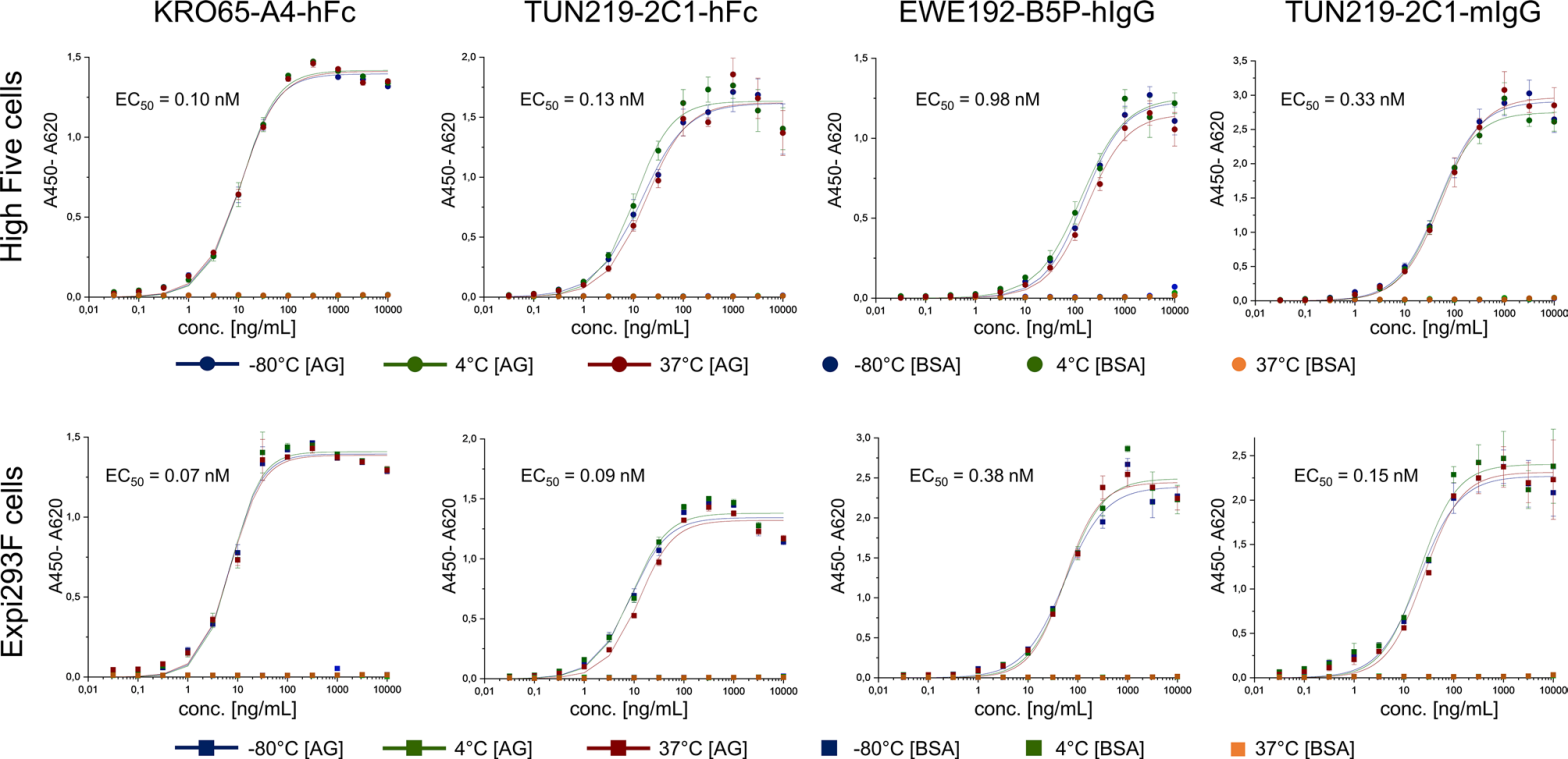
ELISA activity of four different antibodies produced in High Five cells or in Expi293F cells after 30 days of storage at the indicated temperatures. An average EC_50_ value of the different antibodies is given including all storage temperatures. Triplicates were measured, the standard deviation is indicated and, unspecific binding to BSA was tested.

Mammalian expression systems are well established to produce large amounts of antibodies. Antibodies produced in insect cells will have a different glycosylation pattern and therefore might have slightly different characteristics regarding stability and activity. In that respect, we compared four antibodies produced either in Expi293F or in High Five cells. Functionality was assessed by ELISA (Fig. 4) after storage for 30 days at different temperatures (− 80 °C, 4 °C and 37 °C).

**Figure 5:**
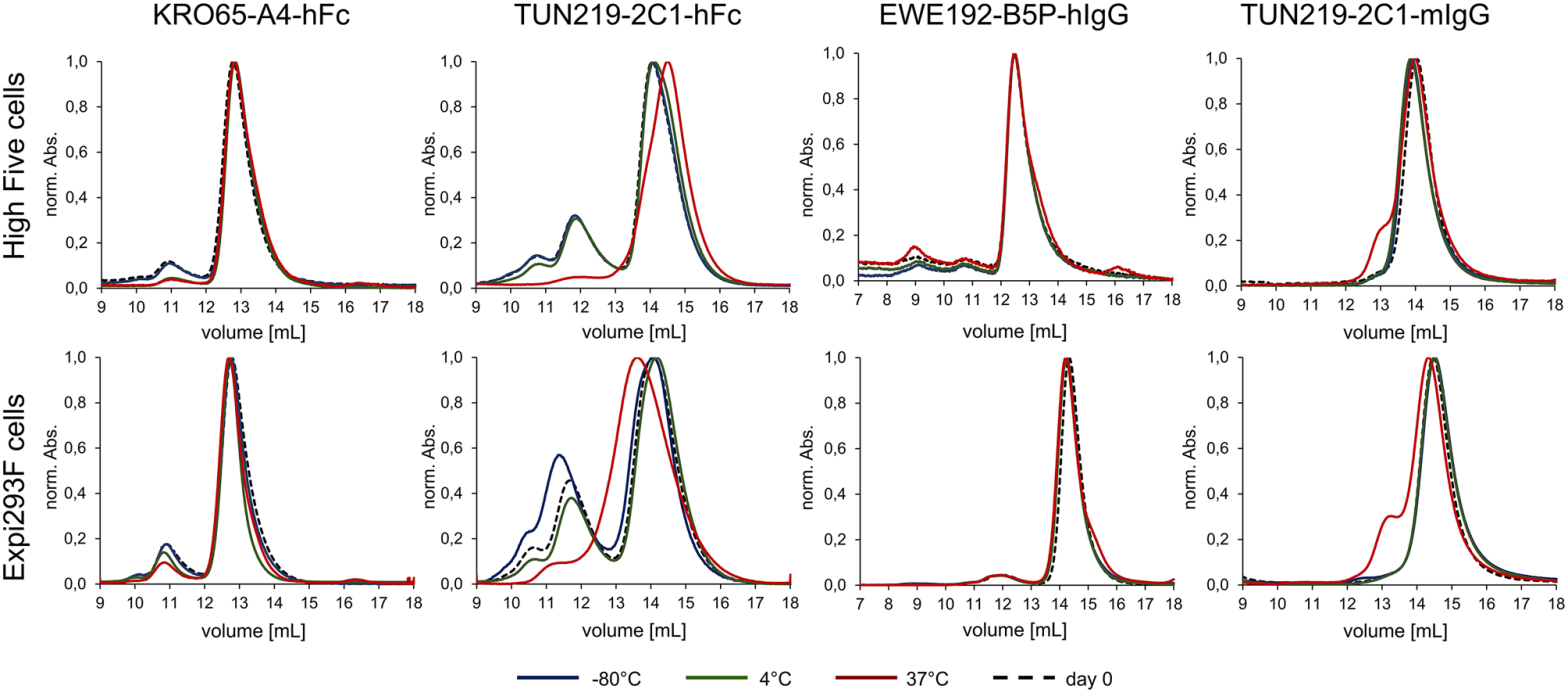
Analytical SEC profile to access aggregation behaviour. The indicated four different antibodies produced in High Five cells or in Expi293F cells were analysed on SEC immediately after production and after 30 days of storage at the indicated temperatures.

No significant differences in antigen binding activity were observed for the four tested antibodies, not even when stored at the different conditions (Fig. 4). Only a slight tendency to a higher EC_50_ value when produced in High Five cells was detected. This might be caused by a different aggregation behaviour as aggregates are known to influence ELISA results. This aggregation behaviour could indeed be observed during SEC analysis (Fig. 5). Most of the High Five expressed antibodies (Fig. 5, upper row) did show a lower aggregation compared to antibodies produced in Expi293F cells (Fig. 5, lower row) with exception of the EWE192-B5P-hIgG, where aggregation was comparable. Storage at the different temperatures for 30 days affected antibodies produced in High Five cells as well as antibodies produced in Expi293F cells similarly but with a slight tendency to affect Expi293F produced antibodies more at least for TUN219-2C1-hFc and TUN219-2C1-mIgG.

In summary, while certainly dependent on the individual antibody sequence, it seems that antibodies produced in High Five cells might have a lower tendency to aggregate compared to those from Expi293F cells.

### Lyophilization of antibodies produced in High Five

**Figure 6:**
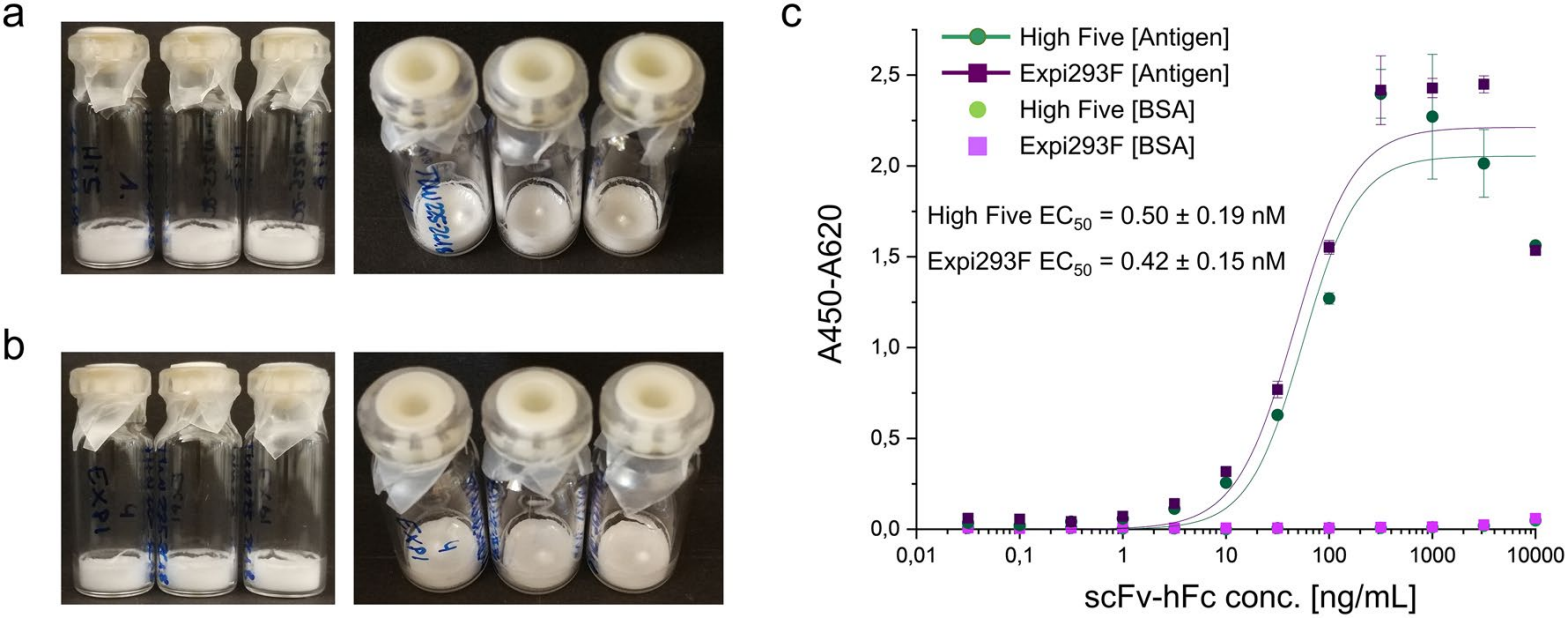
Lyophilization of TUN219-2C1-hFc produced in High Five cells and Expi293F cells. (**a**) Lyophilization cakes of scFv-hFc produced in High Five cells and (**b**) produced in Expi293F cells. (**c**) Antigen ELISA after reconstitution.

Lyophilization is a frequently used stabilization technique for long-term storage and shipment of antibodies. Therefore, it was analyzed whether the expression system influences the lyophilization properties and the activity of one antibody analyzed in this study. Figure 6 shows that neither the cake structure after standard IgG lyophilization nor ELISA activity of antibodies from re-dissolved cakes differed between the scFv-hFc expressed in High Five cells and Expi293F cells.

### Production yields of different secreted proteins in High Five cells compared to Expi293F production

**Figure 7:**
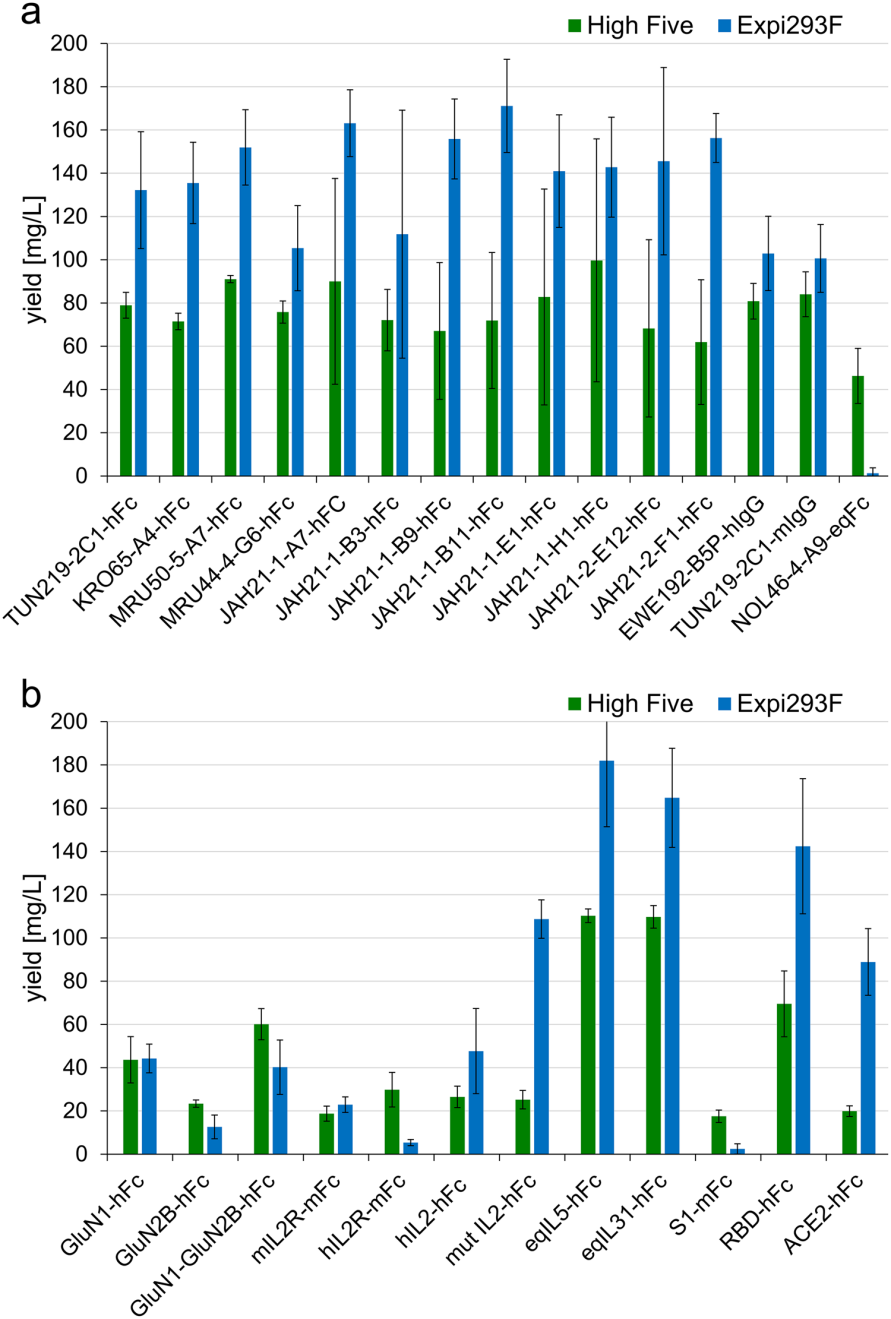
Comparison of the yield of 27 different antibodies and antigens expressed in High Five cells and Expi293F cells. (**a**) 15 different antibodies and (**b**) 12 Fc tagged antigens were expressed in at least four independent experiments in High Five cells (green, 10 mL scale) and in Expi293F cells (blue, 11.5 mL scale). The standard deviation is shown.

To test whether our optimized system is a broadly applicable alternative to established mammalian protein production systems, we compared the yield of 27 different Fc-tagged secreted proteins to our Expi293F cell system (Fig. 7). Hereto, we selected mainly antibodies in scFv-Fc format or as IgG with mouse and human Fc, but also one scFv-Fc with an equine Fc part. In addition, we produced target proteins of the SARS-CoV-2 virus fused to Fc: The S1 and RBD domain of the Spike protein and its receptor ACE2. Furthermore, we decided for a class of difficult-to-express antigens; Interleukins, their receptors and human *N*-methyl-D-aspartate receptors. Detailed information about the antigens is given in Supplementary Data 2.

All 12 tested scFv-hFc reached higher yields in Expi293F cells than in High Five cells by 30% up to nearly 60% (Fig. 7a). In comparison the two tested IgGs had merely a ~ 15–20% higher yield in Expi293F cells then in High Five cells. Most remarkably, the scFv-eqFc was only producible in High Five cells (yield 46 mg/L) but not in Expi293F cells (yield ~ 1 mg/L).

In case of the 12 tested Fc-fused antigens a more diverse expression level was observed (Fig. 7b): Six antigens were produced in higher amounts in Expi293F cells, four in higher amounts in High Five cells and two reached approximately the same amount. Interestingly, a point mutation inserted in IL2-hFc, successfully increasing yields in Expi293F cells^33^ (compare IL2-hFc and mutIL2hFc), did not influence the yield in High Five cells. Strikingly, all antigens were producible in High Five cells in a sufficient amount, whereas in Expi293F cells expression of hIL2R-mFc and S1-mFc failed, demonstrating that for some target proteins the insect cell expression system is better suited.

## Discussion

In this study we could again highlight the significance of the expression vector to gain high yields in the baculovirus- free High Five expression system. We already showed the importance of the promoter (OpiE2) and surrounding enhancing sequences from the polH region in the expression vector to gain optimal results^29^. Interestingly, the 6-base nucleotide sequence upstream of the ATG start codon is revealed to be crucial for protein expression in High Five cells. This is already known for mammalian expression and the most optimal sequence here has been determined to be a GCC(A/G)CCatgG motif, also called Kozak sequence^34^. In contrast, in Sf9 cells combined with the Baculovirus expression system no effect of the 5′UTR was observed^35^. However, nothing was known so far about the influence of the 5′UTR in High Five cells. A more detailed analysis should be conducted to verify the Kozak sequence as optimal sequence in High Five cells. In that respect, also more different signal peptides, including insect cell endogenous signal peptides, should be analyzed. A further optimized vector might improve the High Five cell system even more.

As time point to harvest secreted proteins 96 h was deemed optimal compared to 72 h of expression for intracellular target proteins. Longer expression seemed to improve yields slightly but was not feasible due to the low viability and high contamination by host cell proteins. Another feeding step with the limiting nutrients at a later time point then 48 h might enhance the viability. However, this additional step will at the same time increase costs and effort.

In general, EX-CELL405 media alone seemed to lead to the most optimal results without the need of any additives, contributing to simple handling of the system. In addition, the time window of when to feed the cells after transfection for the first time was quite wide ranging from 4 h to up to 24 h. It could be hypothesized that, if the cells had been diluted earlier, the DNA-PEI complexes had less time to be uptaken by the cells, leading to a decreased transfection efficacy. Feeding with fresh media after 24 h lead to a relatively high standard deviation, possibly related to variable exhaustion of the media caused by the high cell densities. Thus, feeding after 4–24 h keeps the optimal balance between transfection efficacy and cell growth.

A very interesting outcome of our study is that antibodies produced in High Five cells seem to be less prone to aggregation. Supporting this hypothesis are the results of the expression of IL2. Here the point mutation, K35E^33^, leading to reduced aggregation and higher production yields in Expi293F cells, did not influence the yield in High Five cells, hinting that not aggregation is a bottleneck in High Five production. The observed lower aggregation of the antibodies is probably due to their simpler and more homogenous glycosylation pattern. This observation underlines the suitability of the High Five cells system to produce stable diagnostic antibodies with comparable functionality and aptness for lyophilisation to that of Expi293F produced antibodies.

In regard of yield, not only the yield per liter is important but also the yield per costs, since the prices of expression media differ vastly. In our case Expi293F cell media (~ 123.4 €/L) is ~ 35% more expensive than our High Five cell media (90.4 €/L) calculated by the list prices without considering the costs for Glutamine, Pluronic F68 and CO_2_ aeration required for Expi293F cells. Thus, production of both IgGs and production of 6 out of 12 antigens was cheaper in High Five cells emphasizing the capability of the High Five cell system as a competing expression system. In addition, cultivation of High Five cells does not require a CO_2_ aerated shaking device what simplifies the implementation of this expression system compared to a mammalian expression system.

Furthermore, it might be speculated that the 10 mL scale in 30 mL tubes used in the comparison experiments was not optimal for High Five cells in respect of oxygen transfer and/or shear stress and therefore yield. Also we did not observe differences in regard of cell number, glucose/lactate concentration or viability in tubes (10 mL scale) compared to culture vessels (30 mL scale) (Fig. 2). For instance the yield of RBD-hFc had been by ~ 30% (90 mg/L^36^ instead of 70 mg/L) and of S1-mFc even by ~ 170% (36 mg/L^36^ instead of 13 mg/L) higher in large scale (120 mL in 500 mL culture vessel) than here in 10 mL scale. In general, scalability of the system needs to be further investigated but should be possible in both directions after careful evaluation of oxygen transfer and shear stress.

Another main point in favour of the High Five cell system was its ability to produce all here tested proteins in sufficient amounts. In contrast, expression by Expi293F cells failed completely in case of the scFv-eqFc, hIL2R-mFc and S1-mFc. Thus, our High Five cell expression system is a possibility to access former difficult-to-express target proteins.

In conclusion, the plasmid-based, baculovirus-free High Five cell expression system can be regarded as an alternative system to the Expi293F cell expression system or might even be a possible replacement—especially in regard of stable diagnostic antibodies and the difficult-to-express target proteins shown in this study.

## Methods

### Construction of the expression vectors

Variants of the described OpiE2-eGFP^37^ expression plasmid were constructed for expression in High Five cells. This vector was modified to include the described signal peptides, 5′UTRs, other restriction sites and the indicated inserts by common cloning techniques. The vector backbone was not modified and contains the OpiE2 promoter, the IE1 terminator and surrounding enhancing regions of the polH region (ORF 603 and part of ORF1629).

In Expi293F cells pCSE2.6 and pCSE2.7^38^ expression vectors including hFc-tag, mFc-tag, eqFc, constant regions of light and heavy chain of hIgG or mIgG were used^39^, in which the indicated scFv or antigens were inserted by common cloning techniques.

### Recombinant protein expression in High Five cells

If not otherwise indicted, High Five cells (Thermo Fisher Scientific) were grown in EX-CELL405 media (Merck) at 27 °C and 110 rpm. They were passaged every 2–3 days to a cell density of 0.3–0.5 × 10^6^ cells/mL to maintain optimal growth conditions. The cell line used here was passaged for at least 100 times (see Bleckmann et al.^25^) and had a viability of above 95%. If not indicated otherwise, on day of transfection, the cell number was adjusted to 4 × 10^6^ cells/mL by centrifugation at 180×*g* and 4 min and resuspension in fresh media. 1 µg plasmid DNA per 1 × 10^6^ cells was added directly to the cells immediately followed by the addition of 4 µg 40 kDa Polyethylenimine (PEI40, 1 mg/mL stock solution in H_2_O, Polysciences) per 1 × 10^6^ cells. After a minimum cultivation of 4 h up to a maximum of 24 h, three times of the original volume EX-CELL405 media was added. Approximately 48 h after transfection the volume was again doubled adding EX-CELL405 media. Five days after transfection the supernatant was harvested by a two step centrifugation: First 4 min at 180×*g* to remove cells without disrupting them, then 20 min full-speed (< 1500×*g*) to remove smaller cell debris and molecules.

### Recombinant protein expression in Expi293F cells

Recombinant protein expression in Expi293F cells was performed as described before in Bertoglio et al.^36^. In brief, Expi293F cells (Thermo Fisher Scientific) were cultivated in Gibco FreeStyle F17 expression media (Thermo Fisher Scientific) supplemented with 8 mM Glutamine and 0.1% Pluronic F68 (PAN Biotech) at 37 °C, 110 rpm and 5% CO_2._ At the day of transfection, viability was controlled to be above 90% with cell densities between 1.5 and 2 × 10^6^ cells/mL. For formation of DNA:PEI complexes, 1 µg DNA/mL transfection volume and 5 µg of 40 kDa PEI (Polysciences) were first diluted separately in 5% transfection volume in supplemented FreeStyle F17 media. In a second step DNA and PEI were mixed and incubated ~ 25 min at room temperature (RT) before addition to the cells. Two days after the transfection the culture volume was doubled by feeding HyClone SFM4Transfx-293 media (Cytiva) supplemented with 8 mM Glutamine. In addition, HyClone Cell Boost 6 supplement (Cytiva) was added with 10% of the end volume. One week after transfection supernatant was harvested by 15 min centrifugation at 1500×*g*.

### Glucose and Lactate measurement

Samples of the culture were centrifuged first 4 min at 180×*g*, followed by centrifugation for 20 min at 13,000×*g* and the supernatant was analysed with the BIOSEN C-line (EKF Diagnostic) apparatus according to the manufacturer protocol.

### ELISA (enzyme linked immunosorbent assay)

ELISA was performed as described before in Bertoglio et al.^36^. In brief, 100 ng of antigen was immobilized per well on 96 well microtiter plates (MaxiSorp, Thermo Fisher Scientific) in PBS (pH 7.4) overnight at 4 °C. After coating, the wells were blocked with 2% MPBST for 1 h at RT, followed by three washing steps with H_2_O + 0.05% Tween20. The tested IgG or scFv-Fc were added and incubated for ~ 1.5 h at RT. Following another washing step, bound IgG or scFv-Fc were detected by a goat anti-mouse IgG (Fc-specific) conjugated to horseradish peroxidase (A0168, Sigma, 1:42,000 dilution in 2% MPBST) or goat-anti-hIgG(Fc)-HRP (A0170, Sigma, 1: 70,000 dilution in 2% MPBST) depending on the Fc part used in the fusion construct. Visualization took place by addition of 100 µL/well tetramethylbenzidine (TMB) substrate (20 parts TMB solution A (30 mM Potassium citrate; 1% (w/v) Citric acid (pH 4.1)) and 1 part TMB solution B (10 mM TMB; 10% (v/v) Acetone; 90% (v/v) Ethanol; 80 mM H_2_O_2_ (30%))). After stopping the reaction by addition of 1 N H_2_SO_4_, absorbance at 450 nm with a 620 nm reference was measured in an ELISA plate reader (Epoch, BioTek). Data were analyzed by OriginPro 2019b (Lehre), using Hill function for EC_50_ calculation.

### Protein A purification

All protein yields shown in this study were determined after protein A purification. MabSure Select (Cytiva) or Praesto Jetted A50 (Purolite Life Sciences) Protein A resin was used for affinity enrichment of the Fc fusion proteins. First, binding buffer according to the supplier was added to the centrifuged supernatant and it was filtered using a 0.2 µm filter. Small scale purification (10–40 mL) was done manually in 24 well filter plates with 0.5 mL resin according to the manufactures protocol. Large scale purifications were done using Äkta (Cytiva) or Profinia (BIO-RAD) according to the manufacturers protocol. All antibodies were directly re-buffered to PBS manually using 5 mL Zeba Spin Desalting column (Thermo Fisher Scientific) or 5 mL HiTrap Desalting column (Cytiva).

### SDS–PAGE (SDS–polyacrylamide gel electrophoresis)

SDS–PAGE was performed following the known standard procedures. A 12% separating gel was used and samples were heated in β-mercaptoethanol containing sample buffer at 95 °C for 5 min before loading.

### Analytical size exclusion chromatography

For analytical SEC, antibodies and scFv-Fc were run on Superdex 200 Increase 10/300GL (Cytiva) on Äkta systems following the manufacturers instructions.

### Lyophilization

In brief, 180 µL of scFv-Fc in concentration of 0.525 mg/mL in 0.01 M sodium phosphate buffer (pH 7.4) + 9 µL of a 40% Trehalose solution was lyophilized in 2 mL crimp neck vials (ND 11, 12 × 32 mm) in the Martin Christ Alpha 2–4 LSCplus device. Samples were pre-frozen to − 80 °C (actual self temperature − 65 °C) and a primary drying took place at 0.1 mbar at − 50 °C for 30 min followed by − 35 °C for ~ 37 h. The secondary drying was performed for 32 h at 0.08 mbar at 20 °C. The vials were stored at − 80 °C before testing in ELISA.

## Supplementary Information

Supplementary Information.

## Data Availability

The data that support the findings of this study and is not already shown is available from the corresponding author upon reasonable request.
