# Supplementary material for: Baculovirus-free insect cell expression system for high yield antibody and antigen production
Source: Sci Rep. 2020 Dec 7;10:21393. doi: 10.1038/s41598-020-78425-9 (PMC7721901; doi:10.1038/s41598-020-78425-9)
Supplement: Supplementary file 1 — Supplementary Information. [file 41598_2020_78425_MOESM1_ESM.docx]

**Supplementary information**

**Baculovirus- free insect cell expression system for high yield antibody and antigen production**

Janin Korn^1^, Dorina Schäckermann^1^, Toni Kirmann^1,2^, Federico Bertoglio^1^, Stephan Steinke^1^, Janyn Heisig^1,3^, Maximilian Ruschig^1^, Gertrudis Rojas^4^, Nora Langreder^1^, Esther Veronika Wenzel^1^, Kristian Daniel Ralph Roth^1^, Marlies Becker^1^, Doris Meier^1^, Joop van den Heuvel^5^, Michael Hust^1^, Stefan Dübel^1^, Maren Schubert^1^*

^1^Technische Universität Braunschweig, Department of Biotechnology, Spielmannstraße 7, 38106 Braunschweig, Germany

^2^present address: Universität Leipzig, Medical Faculty, Carl Ludwig Institute for Physiology, Liebigstraße 27, 04103 Leipzig, Germany.

^3^present address: Helmholtz Centre for Infection Research, Department Vaccinology and Applied Microbiology, Inhoffenstraße 7, 38124 Braunschweig, Germany

^4^Center of Molecular Immunology, Havana 11300, PO Box 16040, Cuba

^5^Helmholtz-Centre for Infection Research, Department Structure and Function of Proteins, Inhoffenstraße 7, 38124 Braunschweig, Germany

*author for correspondence, Email: maren.schubert@tu-bs.de

**Supplementary Data 1: Original SDS-PAGEs (not grouped, not cropped)**

**a Supernatants of KRO65-A4-hFc production (10 mL scale production)**

**
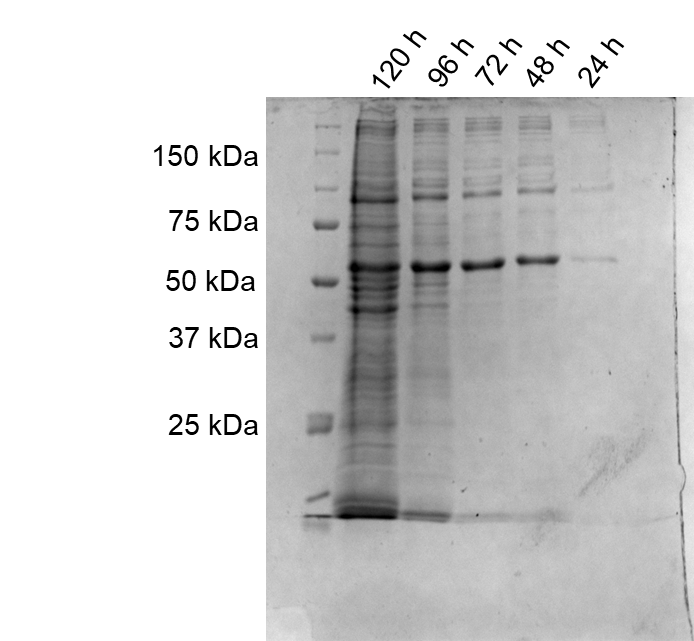
**

**b Supernatants of KRO65-A4-hFc production (30 mL scale production)**

**
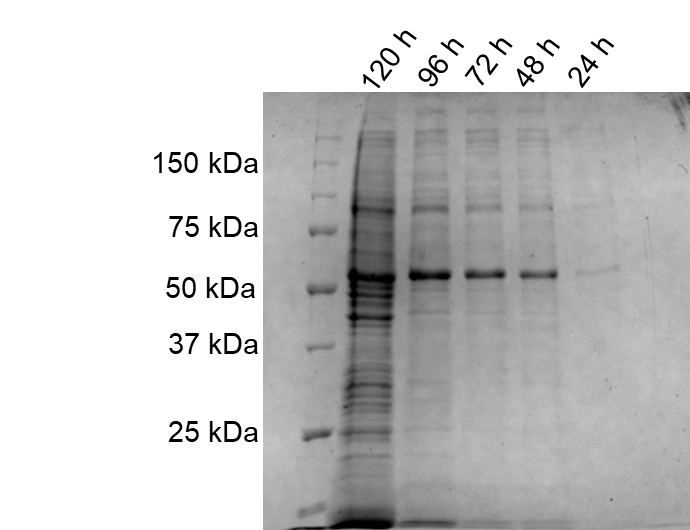
**

**c Supernatants of TUN219-2C1-mIgG production (production scale as indicated)**

**
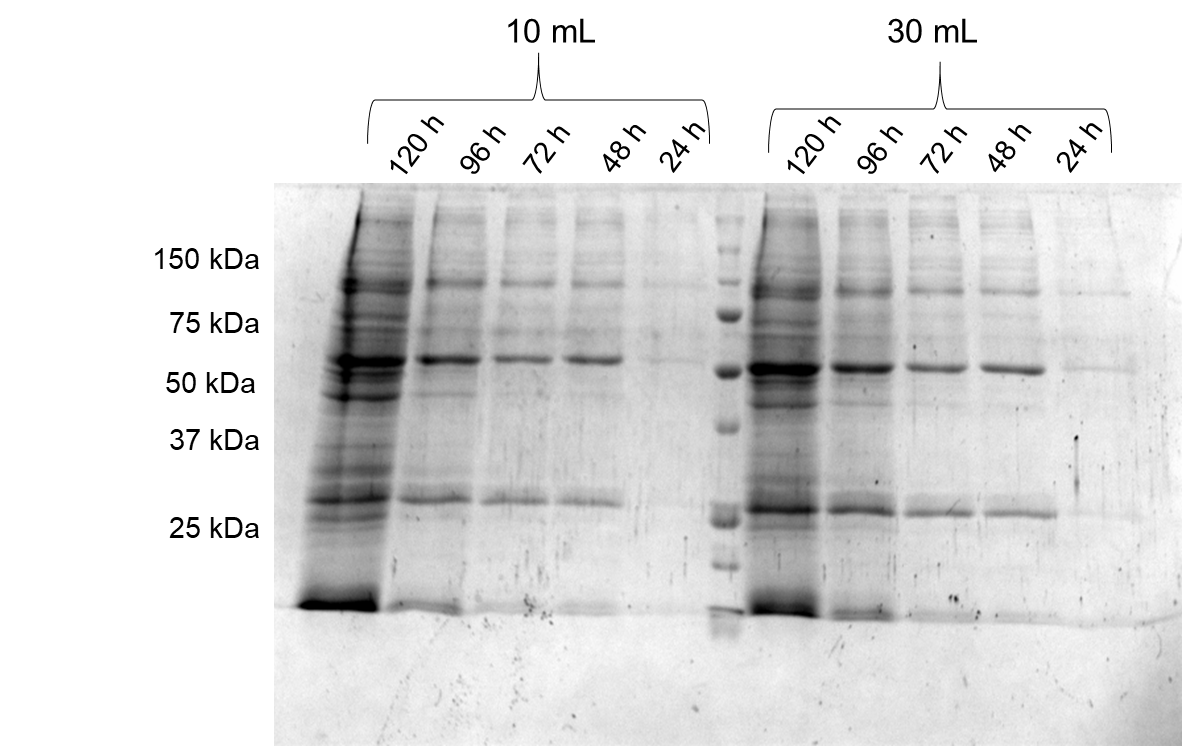
**

**Supplementary Data 2: Overview of the produced antigens and their characteristics**

**hIL2** (human inflammatory Interleukin-2) is a 133 aa member of the four alpha-helix budle cytokine familiy

**muthIL2** is a single mutated (K35E) engineered variant of hIL-2 (Rojas *et al*, 2019)

**hIL2R** (human inflammatory Interleukin-2-receptor beta) comprises 213 aa of the extracellular domain (A27-D239) of human IL-2 receptor beta chain, a membrane protein belonging to type I cytokine receptor family and containing fibronectin type-III structural motifs

**mIL2R** (mouse inflammatory Interleukin-2-receptor beta) comprises 214 aa of the extracellular domain (A27-D240) of mouse IL-2 receptor beta chain, a membrane protein belonging to type I cytokine receptor family and containing fibronectin type-III structural motifs

**eqIL5** (horse inflammatory Interleukin-5) is a proinflammatory cytokine that plays a key role in the differentiation, activation, and effector function of eosinophils.

**eqIL31** (horse inflammatory Interleukin-31) belongs to the gp130/IL‐6 cytokine family and is associated with cellular immunity against pathogens and numerous chronic inflammatory diseases.

**RBD** is the receptor-binding-domain of the SARS-CoV-2 Spike protein (aa319-591).

**S1** is a domain of the SARS-CoV-2 spike protein (aa14-694) containing the RBD and a mutated Furin site according to Wrapp *et al.* 2020.

**GluN1** and **GluN2B** are different subtypes of *N*-methyl-D-aspartate receptors (NMDARs). They are ionotropic glutamate receptors that play an essential role in mediating excitatory neurotransmission in the mammalian central nervous system (CNS) and occur as heterodimer.

**ACE2** (human Angiotensin I converting enzyme 2) is the receptor of SARS-CoV-2 spike protein and its extracellular domain (aa 18-805) was here considered for expression.
